# Brown and white adipose tissues: intrinsic differences in gene expression and response to cold exposure in mice

**DOI:** 10.1152/ajpendo.00473.2013

**Published:** 2014-02-18

**Authors:** Meritxell Rosell, Myrsini Kaforou, Andrea Frontini, Anthony Okolo, Yi-Wah Chan, Evanthia Nikolopoulou, Steven Millership, Matthew E. Fenech, David MacIntyre, Jeremy O. Turner, Jonathan D. Moore, Edith Blackburn, William J. Gullick, Saverio Cinti, Giovanni Montana, Malcolm G. Parker, Mark Christian

**Affiliations:** ^1^Institute of Reproductive and Developmental Biology, Department of Surgery and Cancer, Imperial College London, London, United Kingdom;; ^2^Section of Paediatrics, Division of Infectious Diseases, Department of Medicine, Imperial College London, London, United Kingdom;; ^3^Department of Experimental and Clinical Medicine, Obesity Center, United Hospitals-University of Ancona (Politecnica delle Marche), Ancona, Italy;; ^4^Warwick Systems Biology Centre, University of Warwick, Coventry, United Kingdom;; ^5^Metabolic Signalling Group, Medical Research Council Clinical Sciences Centre, Imperial College London, London, United Kingdom;; ^6^Norwich Medical School, University of East Anglia, Norwich, United Kingdom;; ^7^School of Biosciences at the University of Kent, Canterbury, Kent, United Kingdom;; ^8^Department of Mathematics, Statistics Section, Imperial College London, London, United Kingdom; and; ^9^Division of Metabolic and Vascular Health, Warwick Medical School, University of Warwick, Coventry, United Kingdom

**Keywords:** brown adipose tissue, subcutaneous white adipose tissue, visceral white adipose tissue, cold, brite, adipokine

## Abstract

Brown adipocytes dissipate energy, whereas white adipocytes are an energy storage site. We explored the plasticity of different white adipose tissue depots in acquiring a brown phenotype by cold exposure. By comparing cold-induced genes in white fat to those enriched in brown compared with white fat, at thermoneutrality we defined a “brite” transcription signature. We identified the genes, pathways, and promoter regulatory motifs associated with “browning,” as these represent novel targets for understanding this process. For example, neuregulin 4 was more highly expressed in brown adipose tissue and upregulated in white fat upon cold exposure, and cell studies showed that it is a neurite outgrowth-promoting adipokine, indicative of a role in increasing adipose tissue innervation in response to cold. A cell culture system that allows us to reproduce the differential properties of the discrete adipose depots was developed to study depot-specific differences at an in vitro level. The key transcriptional events underpinning white adipose tissue to brown transition are important, as they represent an attractive proposition to overcome the detrimental effects associated with metabolic disorders, including obesity and type 2 diabetes.

adipose tissue is a highly dynamic endocrine organ with central roles in the regulation of energy metabolism. Discrete adipose tissue depots have distinct contributions to energy balance, and metabolic disorders are associated with its disturbance (21, 43, 56, 59). The major fat depots in mammals, including humans, are the subcutaneous and the intra-abdominal depots. These are composed of varying amounts of the two different types of adipose tissue: white adipose tissue (WAT) that stores energy in the form of triacylglycerol (TAG) and brown adipose tissue (BAT) that dissipates energy as heat, “burning” fatty acids to maintain body temperature. WAT and BAT have significant transcriptional, secretory, morphological, and metabolic differences. The main murine BAT depot is located subcutaneously in the interscapular region, but it can also be found in the cervical, axillar, and paravertebral regions. BAT is a highly vascularized and innervated tissue, and brown adipocytes possess multilocular lipid droplets and a high number of mitochondria and demonstrate a high rate of fatty acid and glucose uptake and oxidation (9). WAT is found primarily under the skin (subcutaneous) or inside the abdomen (gonadal, mesenteric, omental, and perirenal), and it is much less innervated and vascularized than BAT. White adipocytes have a unilocular lipid droplet, few mitochondria, and a low oxidative rate. Important differences exist between subcutaneous and visceral depots, which support their different involvement in insulin sensitivity and type 2 diabetes, with visceral WAT being more closely associated with an adverse metabolic profile (28).

In recent years, considerable interest has risen in the potential beneficial effects of acquiring BAT features (browning) in nonclassic BAT locations such as WAT. It is known that subcutaneous WAT is more sensitive to acquisition of BAT characteristics than visceral depots in mice and humans (47, 53). However, it is still a matter of debate as to whether mature white adipocytes undergo transdifferentiation into brown adipocytes (12, 16) or whether precursors committed to the brown lineage already present in the tissue differentiate into mature brown adipocytes (42, 63). Brown in white (brite) or “beige” adipocytes are distinct from the adipocytes in interscapular BAT in that they arise as multilocular adipocytes in WAT during adrenergic stimulus (30, 42, 46, 61, 63). They have low levels of uncoupling protein 1 (UCP1) compared with classic brown adipocytes but possess the capacity to induce UCP1 expression to a very high extent and the ability to increase their oxygen consumption upon adrenergic activation. It has been reported recently that the adipocytes found in human BAT could be of this brite type, and a defined bona fide interscapular BAT depot is found in infants (13, 31, 36, 49, 63). Several classes of factors have been identified to promote a BAT phenotype either in white adipocytes and/or WAT. These include transcription factors [e.g., peroxisome proliferator-activated receptor-γ (PPARγ), CCAAT/enhancer-binding protein beta (C/EBPβ), and Foxc2], coregulators [e.g., PPARγ coactivator-1α (PGC-1α), Prdm16, and Plac8], growth factors [e.g., bone morphogenetic protein (Bmp)7, Fgf21, Bdnf, and Bmp8b], enzymes (e.g., Ptgs2), lipid droplet-associated proteins (e.g., perilipin), and cell survival factors (e.g., necdin) as well as the miR193b-365 microRNA cluster (reviewed in Ref. 51). Although these factors have important roles in promoting a brown fat phenotype, to date, only fibroblast growth factor 21 (FGF21) has been reported in a therapeutic study (17). Understanding WAT-BAT plasticity in different depots, it is important for the design of future treatments to target type 2 diabetes and obesity by BAT-inducing drugs or other therapies, such as tissue transplantation or gene therapy.

For this study, we have compared gene expression in four different mouse adipose depots with very specific physiologies upon prolonged cold exposure. We sought to identify differentially regulated genes and thereby novel signaling pathways during cold-induced adipose tissue “remodeling.” Furthermore, we identified motifs that are enriched in the promoters of brite genes. We have been able to confirm that subcutaneous adipose tissue is the most prone to changing to display a brite phenotype identified as a number of temperature-regulated and differentially expressed genes that represent new targets for further studies. In addition, we have established cell models that will allow the comparison of different adipose tissue depots at an in vitro level. From these investigations, we have demonstrated that neuregulin 4 (Nrg4)is a novel cold-induced adipokine that promotes neurite outgrowth.

## MATERIALS AND METHODS

### Mice

#### Cold exposure experiments.

Groups of four 10-wk-old female 129Sv mice (Charles River, Milan, Italy) were housed separately and kept at 22°C. Subsequently, one group of mice was placed at 28°C and another one at 6°C for 10 days. Another group was kept at 22°C. Animals were singly caged with a 12:12-h light-dark cycle and free access to standard chow diet and water. At the end of the treatment, the body weights of single animals did not change significantly either during cold exposure or in warm acclimatization. Mice were culled as reported previously (7), and adipose tissues (interscapular BAT and subcutaneous, mesenteric, and gonadal WAT) were collected. Care and handling were in accordance with institutional guidelines. Procedures were approved by the Ethics Committee for Animal Experiments at the University of Ancona (11 Gen. 2007, protocol no. 585).

#### β_3_-Adrenoceptor agonist administration experiments.

Two groups of four 10-wk-old C57BL/6 female mice kept at 22°C were given an intraperitoneal injection either with saline (200 μl) or CL316,243 (1 mg/kg body wt in 200 μl of saline) 5 h after injection mice were culled by cervical dislocation and adipose tissues collected.

#### Tissue panel.

FVB/N female mice kept under normal housing conditions were culled by cervical dislocation and the different tissues collected. Experiments were carried out in accordance with UK Home Office regulations.

### Immunohistochemistry and Immunocytochemistry

Immediately after removal, adipose tissues were fixed overnight by immersion at 4°C in 4% paraformaldehyde. Paraffin-embedded dewaxed sections were stained with hematoxylin and eosin or incubated with anti-UCP1 (cat. no. 10983; Abcam) or anti-Nrg4 (anti-127; see Ref. 24) according to the avidin-biotin complex method (27). Staining was never observed when the primary antibody was omitted.

### RNA Extraction and qRT-PCR

Total RNA was extracted with Trizol. RNA for microarray analysis was purified using Qiagen RNeasy mini columns. The expression of target genes was determined using SYBR green reagent and gene-specific primers (sequences available on request). Relative expression levels were normalized to β-actin.

### Microarray Analysis

Gene expression microarray was performed by Almac diagnostics UK using GeneChip Mouse Genome 430A 2.0 Array (Affymetrix) on RNA extracted from interscapular BAT and subcutaneous and mesenteric WAT from mice exposed to 28 or 6°C. Three biological replicates were used. The analysis was conducted using R programming language and Bioconductor (55). After background adjustment, summarization, and normalization of the raw intensities, the data were analyzed using affy (19) and limma (14). Pairwise comparisons between conditions were performed. A linear model was fitted to the expression values of each probe, modified to account for the replicated samples and assuming no interaction between the genes. The moderated *t*-statistic was computed for each probe and for each contrast providing *P* values that were corrected for multiple testing using the Benjamini and Hochberg's method to control the false discovery rate (10). Probes with adjusted *P* values of <0.01 were considered statistically significant. The microarray data have been deposited in NCBI's Gene Expression Omnibus (15) and are accessible through GEO Series accession no. GSE51080 (http://www.ncbi.nlm.nih.gov/geo/query/acc.cgi?acc=GSE51080). Heatmaps were produced based on the Euclidian distance. Pathway analysis was generated by Ingenuity IPA software.

### Protein Expression

Tissues and cultured cells were lysed in Laemmli loading buffer, and proteins were electrophoresed on a 10% SDS-PAGE gel and transferred to PVDF membrane. Membranes were probed with specific antibodies UCP1 (cat. no. U6382; Sigma), cell death-inducing DNA fragmentation factor α-subunit-like effector A (CIDEA; cat. no. AB16922; Chemicon), p-Akt (Ser^473^), and t-Akt, all from cell-signaling technologies and β-actin (Sigma).

### Preparation of adipocytes vs. stromal vascular fraction-enriched fractions

Adipose tissues from four mice were cut into 2-mm pieces and digested in serum-free DMEM-F-12 culture medium containing 1 mg/ml collagenase A (Roche) and 10 mg/ml DNAse (Roche). After digestion, the cells were separated by centrifugation at 170 *g* for 10 min, and mature adipocytes (floating fraction) and stromal vascular fraction (SVF; in the cell pellet) were collected separately for RNA extraction.

### Cell Culture

#### Generation of immortalized cell lines.

Primary cultures of brown adipose and subcutaneous, mesenteric, and gonadal white adipose tissues were generated by first digesting the tissue and pelleting the SVF, as described above. Preadipocytes were purified by collecting the cells that passed through 100 μm of mesh. Next, cells were passed through strainers of 70- or 25-μm mesh size for brown and white preadipocytes, respectively, and were plated and cultured in DMEM-F-12, 10% FBS, and 1% antibiotic-antimycotic. After culture for 2 days, preadipocytes were immortalized by retroviral-mediated expression of temperature-sensitive SV40 large T-antigen H-2kb-tsA58 (a gift from P. Jat). Cells were cultured at 33°C and selected with G418 (100 mg/ml) for 2 wk and then maintained in 50 μg/ml G418. Experiments were performed between passages 12 and 22. Cells were differentiated as described previously (22, 39).

#### PC12-HER4.

The PC12-HER4 cell line (24) was cultured in RPMI-1640 supplemented with 10% horse serum and 5% fetal bovine serum. Cells were plated on poly-l-lysine-coated glass coverslips and exposed to unconditioned DMEM-F-12, 10% FBS medium, or conditioned medium from differentiated adipocytes.

### Metabolic Studies

#### 9,10-[3H]palmitate oxidation.

Assessment was performed as reported previously (18). Briefly, differentiated adipocytes were starved in glucose-deprived DMEM for 1 h. Cells were then incubated at 37°C in DMEM containing 0.3 mM palmitate (9,10-[^3^H]palmitate, 12 μCi/ml), 2% BSA, 0.25 mM l-carnitine, and 3 mM glucose for 3 h. Palmitate oxidation was assessed by measuring ^3^H_2_O produced in the incubation medium. Data were normalized to DNA content/well measured with a Fluorescent DNA Quantitation Kit (Bio-Rad).

#### Glucose uptake.

Glucose uptake was measured using 2-[*N*-(7-nitrobenz-2-oxa-1,3-diazol-4-yl)amino]-2-deoxy-d-glucose (2-NBDG; Molecular Probes). Differentiated adipocytes in 96-well plates were serum starved for 3 h. Then cells were incubated in HBSS buffer with or without insulin (100 nM) for 30 min. Glucose uptake was initiated by the addition of HBSS containing 500 μM 2-NBDG and 2.5 mM deoxyglucose. After 15 min, plates were washed with cold HBSS buffer containing 25 mM glucose. Cells were lysed, and fluorescence was read at 466 nm. Fluorescence was normalized to protein content.

### Nrg4 ELISA

NRG4 levels in culture medium were determined by ELISA (SEC147Mu; Caltag Medsystems), following the manufacturer's protocol.

### IL-6 ELISA

Antibodies and standards were sourced from R & D Systems, and the ELISA was performed according to the manufacturer's instructions. Data were normalized to DNA content/well measured with a Fluorescent DNA Quantitation Kit (Bio-Rad).

### Nrg4 Knockdown

Conditionally immortalized BAT cells were transfected with shRNA (short-hairpin RNA) targeting Nrg4 (pSuperior-shNrg4). Oligonucleotides (shNrg4 forward, 5′-GATCCCCUGGGGGGAUUUGUUAUGUGTTCAAGAGACACUAACAAAUCCCCCCATTTTTA-3′; and shNrg4 reverse, 5′-AGCTTAAAAAUGGGGGGAUUUGUUAUGUGTCTCTTGAACACAUAACAAAUCCCCCCAGGG-3′) were annealed and ligated into pSuperior vector. Cells stably expressing the shRNA were achieved by selection with puromycin.

### Statistical Analysis

ANOVA tests were performed, and only *P* values <0.05 considered statistically significant. In all figures, the data are the average of a minimum of three independent replicates ± SE.

### Motif Detection in Upstream Flanking Regions of Differentially Expressed Genes

Lists of differentially expressed genes from the expression experiments were ordered using fold change, and Ensembl Gene IDs were obtained for lists of interest. The Ensembl Gene IDs were subsequently submitted to BioMart (Ensembl Genes 72, Mus musculus GRCm38.p1 dataset) to retrieve the corresponding 1,000-bp upstream flanks of the transcripts of the genes of interest from the genome annotation. To remove sequence repeats arising from overlapping transcript variants, the FASTA sequences were assembled into contiguous sequences with CAP3. Mixed contiguous sequences composed of flanks from more than one gene were verified to identify overlaps generated in CAP3 due to sequence similarity alone rather than contiguity. Such false mixed contiguous sequences were removed from the set of contiguous sequences. The original flanking regions making up the false mixed contiguous sequences were then collected, and a contiguous sequence was rebuilt if there was more than one transcript-flanking region for a particular gene, whereas singlets were simply added to the final set. The final set of sequences was then collated, which included the initial true contiguous sequences, the singlets, and the singlets or contiguous sequences which arose from the false mixed contiguous sequences. The resulting FASTA sequence file was submitted as a single cluster to MEME-LaB to detect 10 motifs between six and 12 base pairs, with the promoter maximum length adjusted to accommodate the longer contiguous sequences.

## RESULTS

### Fat Depots Vary in Their Response to Cold

**Fig. 1.**
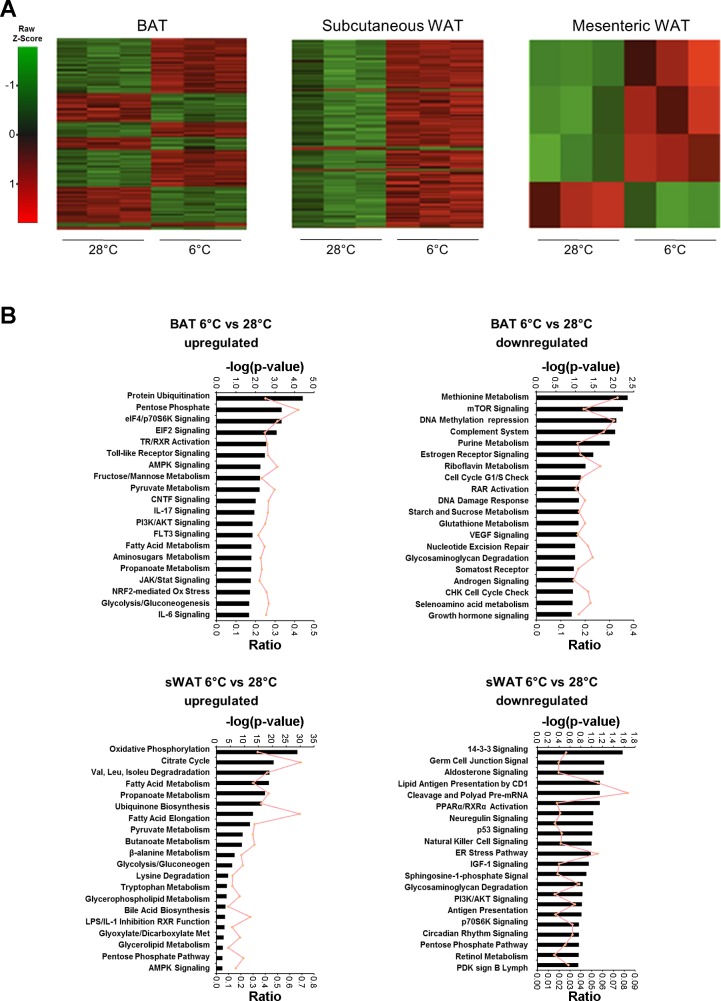
Global expression analysis of brown adipose tissue (BAT) and subcutaneous and mesenteric white adipose tissue (WAT) in mice exposed to cold. *A*: global mRNA expression was measured in BAT and subcutaneous and mesenteric WAT depots of mice exposed at either 28 or 6°C using Affymetix 2.0 ST microarrays. A row-centred heat map of hierarchical clustering carried out on the differentially expressed gene probes at a 5% false discovery rate is shown. Probe sets are colored according to the average expression level across all samples, with green denoting a lower expression level and red denoting a higher expression level. *B*: top pathways that were altered by cold exposure in BAT and subcutaneous WAT. The bars represent significance, and the ratio of genes altered out of the total genes per pathway is indicated. Pathway analysis was performed using up- and downregulated probes separately. mTOR, mammalian target of rapamycin; EIF, eukaryotic initiation factor; NRF2, nuclear factor-E2-related factor; PI3K, phosphatidylinositol 3-kinase; CNTF, ciliary neurotrophic factor; RAR, retinoic acid receptor.

To study the ability of different adipose tissue depots to respond to changes in temperature, 129Sv female mice were exposed to temperatures of 28 or 6°C. This mouse strain is reported to be diabetes and obesity resistant due to a greater amount of active BAT (3). It has a greater amount of adipocytes expressing UCP1 in different adipose depots and has been used for several studies addressing cold-induced transdifferentiation of adipose tissues (7, 40). Global changes in expression were monitored by microarray analysis of BAT and inguinal subcutaneous and mesenteric WAT. BAT showed the most dramatic changes upon prolonged cold exposure, with 11,180 gene probes sets differentially expressed (*P* < 0.01) (Fig. 1*A*). In subcutaneous WAT, 2,178 probes were altered, but only eight were altered in mesenteric WAT.

By considering the up- and downregulated probes separately, we identified the most significantly differentially expressed pathways in each tissue. The top 20 pathways for BAT and subcutaneous WAT are shown in Fig. 1*B*. The number of genes altered in the mesenteric depot was too low to be used for pathway analysis. In BAT, the top upregulated pathways involve protein degradation (protein ubiquitination), metabolism (fructose, mannose, fatty acid, glucose, and propanoate metabolism), reduced power generation (pentose phosphate pathway), signal transduction [thyroid hormone receptor/retinoid X receptor regulation, Toll-like receptor (TLR) activation, AMPK, IL-17, PI3K/Akt], and oxidative stress regulation. Among the downregulated probes, the most significantly altered pathways are cell cycle- and growth regulation-related pathways (purine metabolism, DNA methylation, cell cycle G_1_/S checkpoint, DNA damage repair) as well as signaling pathways (mTOR, estrogen, RAR, androgen, and VEGF signaling). On analysis of the subcutaneous data set, it becomes evident that this particular depot shows a shift toward a brown or brite phenotype when animals are exposed to 6°C. Most of the top upregulated pathways are those that are highly expressed in BAT (oxidative phosphorylation, citrate cycle, fatty acid metabolism, ubiquinone synthesis, pyruvate metabolism). Downregulated probes belong mostly to pathways related to the immune system (lipid antigen presentation, natural killer cell signaling, antigen presentation), signaling, and endoplasmic reticulum stress.

### Intrinsic Differences of WAT and BAT Depots at Thermoneutrality

**Fig. 2.**
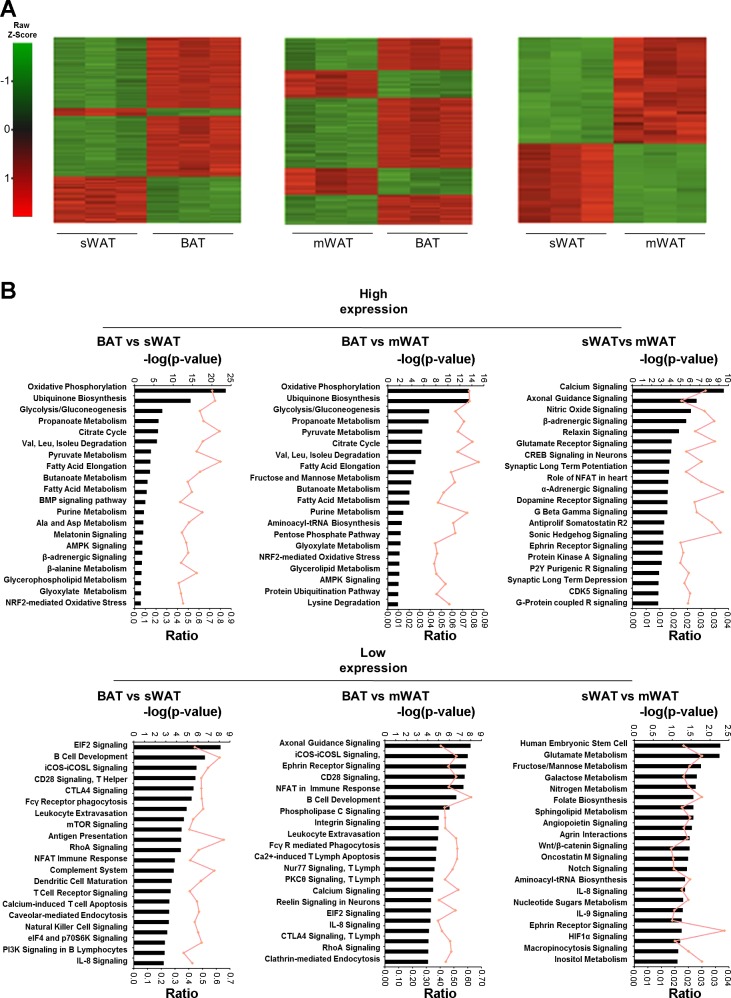
Global expression comparison of BAT and subcutaneous (sWAT) and mesenteric WAT (mWAT) at 28°C. *A*: global mRNA expression was measured in BAT, sWAT, and mWAT depots of mice housed at 28°C, using Affymetix 2.0 ST microarrays. Shown is a row-centred heat map of hierarchical clustering carried out on the differentially expressed gene probes at a 10% false discovery rate. Probe sets are colored according to the average expression level across all samples, with green denoting a lower expression level and red denoting a higher expression level. *B*: top pathways that are enriched in each of the different depots compared with each other. The bars represent significance, whereas ratio shows the genes altered out of the total genes per pathway. Pathway analysis was performed using up- and downregulated probes separately.

Using the microarray data, we analyzed the intrinsic differences between each adipose depot at 28°C. In Fig. 2*A*, the heat maps for each of the comparisons are shown. Comparison of BAT to subcutaneous and mesenteric WAT indicated that 25,625 and 30,242 probes, respectively, were differentially expressed, whereas only 9,647 probes were differentially expressed for subcutaneous vs. mesenteric. As expected, these data show that WAT depots are more similar to each other than to BAT.

The most highly expressed pathways in BAT were those involved in energy expenditure, metabolism, and signaling (Fig. 2*B*), with those enriched in WAT related to the immune system, including T cell activation, antigen presentation, complement system activation, and lymphocyte apoptosis. When comparing subcutaneous to mesenteric WAT, the enriched pathways in subcutaneous WAT are related to neuronal signaling, whereas the most prominent pathways in mesenteric WAT seem to be related to interleukin production and signaling as well as carbohydrate and amino acid metabolism.

### Identification of Differentially Expressed Genes Sensitive to Temperature

From the microarray analysis, we aimed to identify genes that were significantly altered in expression by temperature with potential relevance for the temperature-induced acquisition of brite features. The top 10 up- and downregulated genes, due to cold exposure, are presented for BAT and subcutaneous and mesenteric WAT in Table 1, and the top genes for comparisons of the depots at 28°C are presented in Table 2. We chose a set of genes for further study that were more highly expressed in BAT compared with WAT depots and were also increased in subcutaneous WAT following cold exposure. This group included genes involved in several different processes. Nrg4 is a regulator of neuronal development (23), and Bmp8b regulates thermogenesis in BAT (62). The G protein-coupled receptor (GPCR) Gpr120 is linked with obesity (29), and Rgs7 (4) has the potential to affect the signaling through many GPCRs important for BAT function. Slc27a2 (26) and Letm1 (60) have key roles in lipid metabolism and mitochondrial function, respectively. We also identified the uncharacterized transcript 9030619P08RIK for further study, as it is highly regulated and could be a potential cell surface marker of brown adipocytes. This gene shares a high homology with the Ly-6 protein family and is found in the vicinity of a genomic region that contains the genes for this family (6). Hence, we term it cold-induced lymphocyte antigen 6-like (Cil6l). Neuronatin (Nnat), a gene that has been involved in calcium signaling and ER stress and adipose biology (20, 32), was selected because it was downregulated in cold conditions in BAT and subcutaneous WAT.

**Table 1.** Top gene probes up- and downregulated after cold exposure in BAT and subcutaneous and mesenteric WAT

BAT 6°C vs. 28°C Upregulated				BAT 6°C vs. 28°C Downregulated			
Gene Symbol	FC	Adjusted *P* Value	Gene Name	Gene Symbol	FC	Adjusted *P* Value	Gene Name
Elovl3	76.76	<0.001	Elongation of very-long-chain fatty acid-like 3	EG666481	68.85	<0.001	Predicted gene 8126
Bmp8b	32.25	<0.001	Bone morphogenetic protein 8b	1110059M19Rik	37.60	<0.001	RIKEN cDNA 1110059M19
9030619P08Rik	30.40	<0.001	RIKEN cDNA 9030619P08	Snhg11	11.86	<0.001	Small nucleolar RNA host gene 11 (nonprotein coding)
4933433H22Rik	15.12	<0.001	RIKEN cDNA 4933433H22	Myh4	11.67	0.019	Myosin, heavy polypeptide 4
Serpina12	14.88	<0.001	Serine peptidase inhibitor, clade A member 12	Rab17	11.06	<0.001	RAB17 member RAS oncogene family
Gyk	11.83	<0.001	Glycerol kinase	Myh2	10.73	0.014	Myosin heavy polypeptide 2
Ncan	11.71	<0.001	Neurocan	Asb5	10.06	0.005	Ankyrin repeat and SOCS box-containing 5
Rims2	10.38	<0.001	Regulating synaptic membrane exocytosis 2	Ddit4l	9.97	0.016	DNA-damage-inducible transcript 4-like
Slc25a34	9.62	<0.001	Solute carrier family 25, member 34	Casq1	9.89	0.017	Calsequestrin 1
Sgk2	9.51	<0.001	Serum/glucocorticoid regulated kinase 2	B3 galt2	8.52	<0.001	UDP-Gal β-GlcNAc β-1.3-galactosyltransferase, polypeptide 2

Subcutaneous WAT 6°C vs. 28°C Upregulated				Subcutaneous WAT 6°C vs. 28°C Downregulated			
Gene symbol	FC	Adjusted *P* value	Gene name	Gene symbol	FC	Adjusted *P* value	Gene name
Elovl3	31.98	<0.001	Elongation of very-long-chain fatty acid like 3	Csn2	50.46	0.004	Casein-β
Slc27a2	16.64	<0.001	Solute carrier family 27 (fatty acid trasporter) member 2	Nnat	16.72	<0.001	neuronatin
9030619P08Rik	13.29	<0.001	RIKEN cDNA 9030619P08	Kera	5.15	<0.001	Keratocan
Ucp1	12.54	<0.001	Uncoupling protein 1	Defb1	5.06	0.002	Defensin-β1
Fabp3	10.21	<0.001	Fatty acid-binding protein 3	Gm266	4.98	<0.001	predicted gene 266
Cpt1b	9.88	<0.001	Carnitine palmitoyltransferase 1b, muscle	Col9a1	4.17	<0.001	Collagen type IX, α1
Cox7a1	9.21	<0.001	Cytochrome *c* oxidase, subunit VIIa 1	Htr4	4.17	0.004	5 hydroxytryptamine (serotonin) receptor 4
Kng1	7.84	<0.001	Kininogen 1	Slc15a2	3.24	<0.001	Solute carrier family 15 (H+/peptide transporter), member 2
Otop1	7.49	<0.001	Otopetrin 1	Bdnf	3.15	3.93E-03	brain derived neurotrophic factor
Ppara	7.48	<0.001	Peroxisome proliferator-activated receptor-α	Adamts2	3.10	1.70E-04	A disintegrin-like metallopeptidase throm-bospondin type1, motif2

Mesenteric WAT 6°C vs. 28°C Upregulated				Mesenteric WAT 6°C vs. 28°C Downregulated			
Gene symbol	FC	Adjusted *P* value	Gene name	Gene symbol	FC	Adjusted *P* value	Gene name
Ucp1	7.88	<0.001	Uncoupling protein 1	LOC100046232	2.66	<0.001	Nuclear factor interleukin 3, regulated
Orm3	2.84	<0.001	Orosomucoid 3	Tmem182	1.84	<0.001	Transmembrane protein 182
Nrg4	2.18	<0.001	Neuregulin member 4				
Cabc1	2.17	<0.001	Chaperone, ABC1 activity of bc1 complex like				
Acsf2	1.77	<0.001	Acyl-CoA synthetase family member 2				

BAT, brown adipose tissue, WAT, white adipose tissue; FC, fold change.

**Table 2.** Top gene probes up- and downregulated when comparing BAT, subcutaneous WAT, and mesenteric WAT at thermoneutrality

BAT 28°C vs. Subcutaneous 28°C Upregulated				BAT 28°C vs. Subcutaneous 28°C Downregulated			
Gene symbol	FC	Adjusted *P* value	Gene name	Gene symbol	FC	Adjusted *P* value	Gene name
Zic1	111.91	<0.001	Zinc finger protein of the cerebellum 1	Csn3	106.07	<0.001	Casein kappa
Cpn2	77.41	<0.001	Carboxypeptidase N, polypeptide 2	Btn1a1	90.95	<0.001	Butyrophilin, subfamily 1, member A1
Slc27a2	76.77	<0.001	Solute carrier family 27 (fatty acid transporter), member 2	Foxa1	88.56	<0.001	Forkhead box A1
2310014F07Rik	61.34	<0.001	RIKEN cDNA 2310014F07 gene	Areg	82.96	<0.001	amphiregulin
Kng1	53.07	<0.001	Kininogen 1	Ehf	81.38	<0.001	Ets homologous factor
EG666481	44.08	<0.001	Predicted gene 8126	Serpinb5	78.90	<0.001	Serpin peptidase inhibitor, clade B, member 5
Olig1	42.44	<0.001	Oligodendrocyte transcription factor 1	Cxcl15	78.77	<0.001	Chemokine (C-X-C motif) ligand 15
Ppara	40.04	<0.001	Peroxisome proliferator-activated receptor-α	Krt8	75.07	<0.001	keratin 8
9130214F15Rik	38.03	<0.001	RIKEN cDNA 9130214F15 gene	Slc5a5	64.96	<0.001	Solute carrier family 5 (sodium iodide), member 5
Chkb-cpt1b	35.86	<0.001	Carnitine palmitoyltransferase 1b, muscle	Hoxa10	59.03	<0.001	homeobox A10

BAT 28°C vs. Mesenteric 28°C Upregulated				BAT 28°C vs. Mesenteric 28°C Downregulated			
Gene symbol	FC	Adjusted *P* value	Gene name	Gene symbol	FC	Adjusted *P* value	Gene name
Ucp1	210.01	<0.001	Uncoupling protein 1	Upk3b	108.35	<0.001	Uroplakin 3b
Slc27a2	171.79	<0.001	Solute carrier family 27 (fatty acid transporter), member 2	Phox2b	101.69	<0.001	Paired-like homeobox 2b
Zic1	138.04	<0.001	Zinc finger protein of the cerebellum 1	Colec10	95.85	<0.001	Collectin subfamily member 10
2310014F07Rik	124.87	<0.001	RIKEN cDNA 2310014F07 gene	Bnc1	91.40	<0.001	Basonuclin 1
Cpn2	107.34	<0.001	Carboxypeptidase N, polypeptide 2	Scg2	87.39	<0.001	Secretogranin II
Myo5c	65.24	<0.001	myosin VC	Snap25	85.13	<0.001	synaptosomal-associated protein 25
Myh4	64.22	<0.001	Myosin, heavy polypeptide 4, skeletal muscle	Isl1	81.38	<0.001	ISL1 transcription factor, LIM/homeodomain
EG666481	58.31	<0.001	Predicted gene 8126	Rims1	78.79	<0.001	regulating synaptic membrane exocytosis 1
Six1	50.30	<0.001	Sine oculis-related homeobox 1 homolog (Drosophila)	Calcb	77.62	<0.001	calcitonin-related polypeptide, β
Chkb-cpt1b	47.65	<0.001	Carnitine palmitoyltransferase 1b, muscle	Bcl11a	74.34	<0.001	β-cell CLL/lymphoma 11A (zinc finger protein)

Subcutaneous 28°C vs. Mesenteric 28°C Upregulated				Subcutaneus 28°C vs. Mesenteric 28°C Downregulated			
Gene symbol	FC	Adjusted *P* value	Gene name	Gene symbol	FC	Adjusted *P* value	Gene name
Csn3	174.98	<0.001	Casein kappa	Rims1	339.50	<0.001	Regulating synaptic membrane exocytosis 1
Areg	169.91	<0.001	Amphiregulin	Upk3b	263.18	<0.001	Uroplakin 3b
Foxa1	164.17	<0.001	Forkhead box A1	Snap25	235.42	<0.001	Synaptosomal-associated protein 25
Tcfap2c	150.16	<0.001	Transcription factor AP-2, gamma	Scg2	152.72	<0.001	Secretogranin II
Cxcl15	116.61	<0.001	Chemokine (C-X-C motif) ligand 15	Nmu	142.19	<0.001	Neuromedin U
Muc15	113.45	<0.001	mucin 15	Phox2b	131.78	<0.001	Paired-like homeobox 2b
Btn1a1	113.12	<0.001	Butyrophilin, subfamily 1, member A1	Colec10	130.78	<0.001	Collectin sub-family member 10
Elf5	109.85	<0.001	E74-like factor 5	Vip	113.28	<0.001	Vasoactive intestinal polypeptide
Esrp1	98.22	<0.001	Epithelial splicing regulatory protein 1	Gal	99.00	<0.001	galanin
Tmprss2	79.11	<0.001	Transmembrane protease, serine 2	Resp18	95.51	<0.001	Regulated endocrine-specific protein 18

**Fig. 3.**
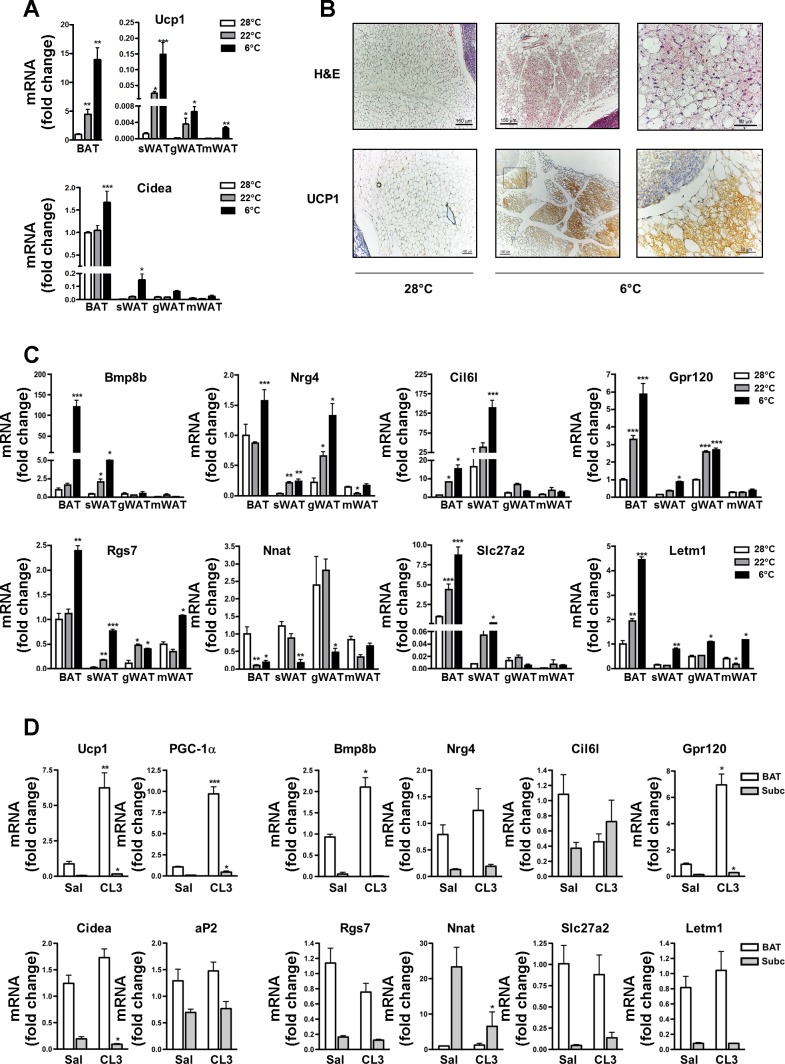
Expression of selected genes induced by cold exposure or by a β_3_-adrenergic-specific agonist in different adipose depots. *A*: mRNA expression of uncoupling protein 1 (UCP1) and cell death-inducing DNA fragmentation factor α-subunit-like effector A (Cidea) in BAT, sWAT, gonadal WAT (gWAT), and mWAT of mice exposed to 28, 22, or 6°C. *B*: hematoxylin and eosin staining (H & E) and UCP1 immunohistochemistry. UCP1-immunoreactive brown adipocytes interspersed among unilocular white adipocytes of subcutaneous adipose tissue of mice exposed at 28 or 6°C. *C*: mRNA expression of genes identified by microarray in BAT, sWAT, gWAT, and mWAT of mice exposed to 28, 22, or 6°C. *D*: mRNA expression of selected genes in different adipose depots of mice injected intraperitoneally with the selective β_3_-receptor agonist CL-316,243 (1 mg/kg body wt) after 5 h; *n* = 4. All mRNA data are expressed as fold induction compared with BAT of mice either at 28°C or injected with saline. Bars represent the means ± SE of at least 3 mice/group, and significant differences are shown. *P < 0.05; **P < 0.005; ***P < 0.001.

Prior to the microarray, we determined the expression of UCP1, a classic cold-induced target gene in BAT by qRT-PCR in the different adipose tissues. We included another visceral fat depot, the gonadal WAT, and tissues from animals housed at 22°C (Fig. 3*A*). UCP1 was induced in all depots after exposure to 6°C compared with either 28 or 22°C, with the subcutaneous depot showing the largest increase. We also measured the expression of Cidea, which was increased in subcutaneous WAT but only modestly in BAT. Hematoyxlin and eosin staining showed a large increase in multilocular cells of the subcutaneous depot upon cold exposure (Fig. 3*B*). Protein expression of UCP1 was monitored in the different depots by immunohistochemistry. Figure 3*B* shows staining of subcutaneous WAT. UCP1 staining increased with exposure to low temperatures in all adipose depots studied (data not shown).

We next validated expression of our genes of interest (Fig. 3*C*). At both 28 and 22°C, Slc27a2 and Letm1 expression was higher in BAT compared with the other depots, and Bmp8b expression was higher in BAT and subcutaneous WAT, whereas Nrg4, GPR120, and Rgs7 were highly expressed in BAT and gonadal WAT depots. In the gonadal depot, the relatively high levels of these genes are likely due to constitutively present brown adipocyte pockets observed in the 129Sv strain even at 28°C (57). Cil6l was expressed largely in subcutaneous WAT, whereas Nnat was preferentially expressed in gonadal WAT, followed by subcutaneous and mesenteric WAT and BAT. Upon cold exposure, we found genes with expression induced in all depots (Rgs7, Letm1), genes with expression induced in BAT and subcutaneous and gonadal WAT (Nrg4, Gpr120), genes induced only in BAT and subcutaneous WAT (Bmp8b, Cil6l, Slc27a2), and a gene that was reduced in all depots (Nnat).

To investigate whether the cold-regulated genes were affected directly by the β_3_-adrenergic signaling pathway, mice were treated with the β_3_-specific adrenergic agonist CL-316,243. Examination of the expression of the cold-responsive genes (Fig. 3*D*, *right*) revealed that only Bmp8b (very modestly) and GPR120 mRNAs were significantly induced by acute adrenergic treatment and only in BAT. These results indicate that many of the genes that are upregulated in WAT result from long-term remodeling effects rather than rapid direct transcriptional events and are likely to be important for brite cell function rather than driving the “browning” process. Alternatively, the genes may initially be refractory to the β_3_-stimulus.

**Fig. 4.**
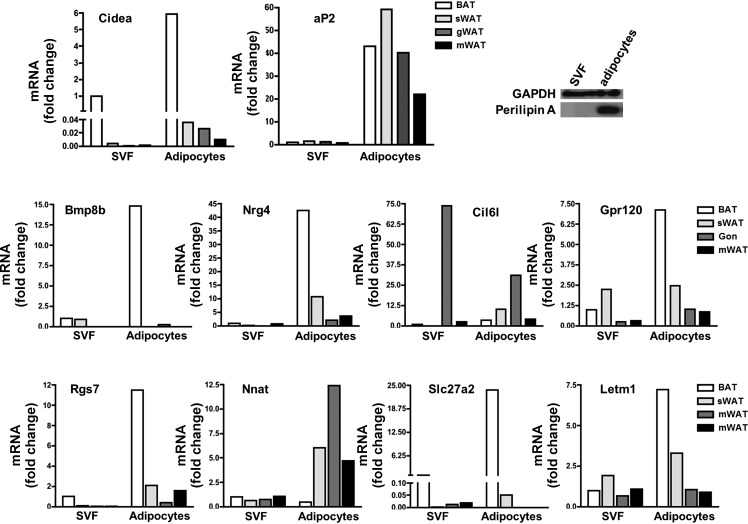
Adipocyte-selective expression of temperature-regulated genes. mRNA expression of in cells from the stromal vascular fraction (SVF) or mature adipocytes prepared from interscapular BAT, sWAT, gWAT (Gon), and mWAT. Perilipin protein levels from gWAT are shown as an indicator of the quality of the fractionation (*top right*). Nrg, neuregulin; Nnat, neuronatin; Cil6l, cold-induced lymphocyte antigen 6-like; Letm1, leucine zipper EF hand-containing transmembrane protein 1.

Because adipose tissues are composed of several cell types, we determined whether the identified genes were expressed in mature adipocytes or nonadipocyte cells that constitute the SVF. Fractionation of the different depots showed that all the genes were preferentially expressed in either brown or white mature adipocytes (Fig. 4).

### Gene Expression in Immortalized Cell Lines Generated From Different Adipose Tissue Depots

**Fig. 5.**
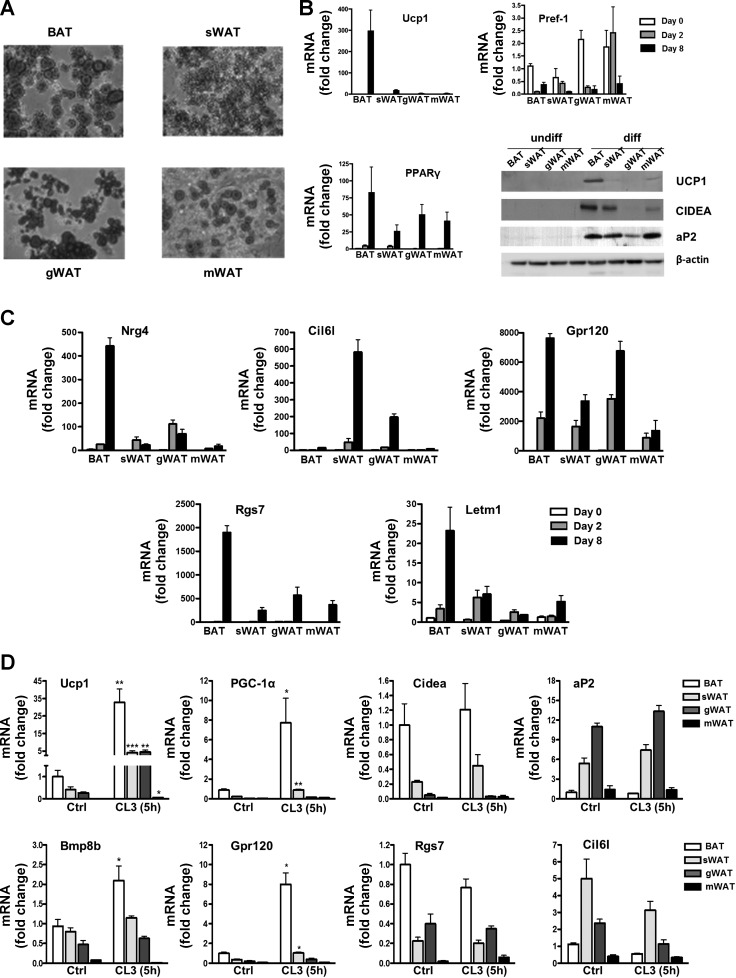
Immortalized cell lines from different adipose tissue depots. *A*: Oil Red O staining of differentiated cells on *days 8–10* of differentiation. *B*: mRNA expression of UCP1, PPARγ, and preadipocyte factor (Pref-1; *n* = 3) and a representative example of Western blot for UCP1, CIDEA, and aP2. *C*: mRNA expression in cells from different immortalized cell lines on *days 0*, *2*, and *8* of differentiation. Data are expressed as fold induction compared with BAT cells on *day 0*; *n* = 3. *D*: mRNA expression in differentiated cells from the different immortalized cell lines that had been treated for 5 h with β_3_-receptor agonist CL-316,243 (10 μM) or vehicle control (Ctrl). Data are expressed as fold induction compared with nonstimulated BAT cells; *n* = 3. For all of the mRNA data, bars represent means ± SE, and significant differences are shown. *P < 0.05; **P < 0.005; ***P < 0.001. PGC-1α, PPARγ coactivator-1α.

Considering that all of the genes we identified were preferentially expressed in mature adipocytes, we next investigated whether their expression was maintained in cells in vitro. We took the approach of immortalizing adipocyte precursors from each adipose depot. This allows the comparison of adipocytes from depots where both large and small numbers of precursors can be obtained, using fewer mice. First, we examined whether the in vitro-differentiated adipocytes retained the features that define each depot. For all the cell lines, 80–100% of cells were differentiated as judged by Oil Red O stain (Fig. 5*A*). The ability to differentiate into adipocytes was maintained after more than 30 passages. At the expression level, the preadipocyte marker Pref-1 and mature adipocyte markers aP2 and PPARγ were downregulated or highly induced, respectively, upon differentiation in the four cell lines (Fig. 5*B*). The brown adipocyte marker UCP1 (Fig. 5*A*) was highly induced in BAT cells after differentiation and to a much lower extent in white adipocyte cells. Expression of UCP1 was almost undetectable in gonadal and mesenteric WAT cells. We then analyzed expression of the identified set of temperature or differentially expressed genes throughout differentiation (Fig. 5*C*). Nnat and Slc27a2 mRNA levels were undetectable at different time points in all cell lines. Expression of the other identified genes was induced upon differentiation to a higher or lower extent in all cell lines. Nrg4, Rgs7, and Letm1 were more highly expressed in differentiated BAT cells compared with the white adipocyte lines. Cil6l was highly expressed in differentiated subcutaneous cells and to a lower extent in gonadal. We next investigated gene regulation due to β_3_-adrenergic signaling. We exposed differentiated adipocytes to an acute (5 h) treatment with CL-316,243. This compound was able to induce UCP1 and PGC-1α mRNA (Fig. 5*D*), showing that the β_3_-adrenergic signaling pathway is fully functional. Regarding the genes identified from the array, we obtained results similar to the in vivo CL-316,243 treatment; GPR120 was the most sensitive to acute adrenergic activation in BAT and subcutaneous cells. In general, Bmp8b was present at low levels in adipocyte cell lines, unlike adipose tissues. However, it was present at higher levels in differentiated brown compared with white adipocytes and was induced by CL-316,243 only in the brown adipocytes (Fig. 5*D*). Nrg4 and Letm1 showed modest induction of mRNA expression in BAT cells and a tendency to increase in subcutaneous cells but not in any of the others. These results highlight that pathways in addition to the β_3_-adrenergic signaling pathway are necessary for the induction of certain genes (Cidea, Nrg4, Rgs7) upon cold exposure, whereas others (UCP1, PGC-1α, GPR120) are regulated directly by this pathway.

**Fig. 6.**
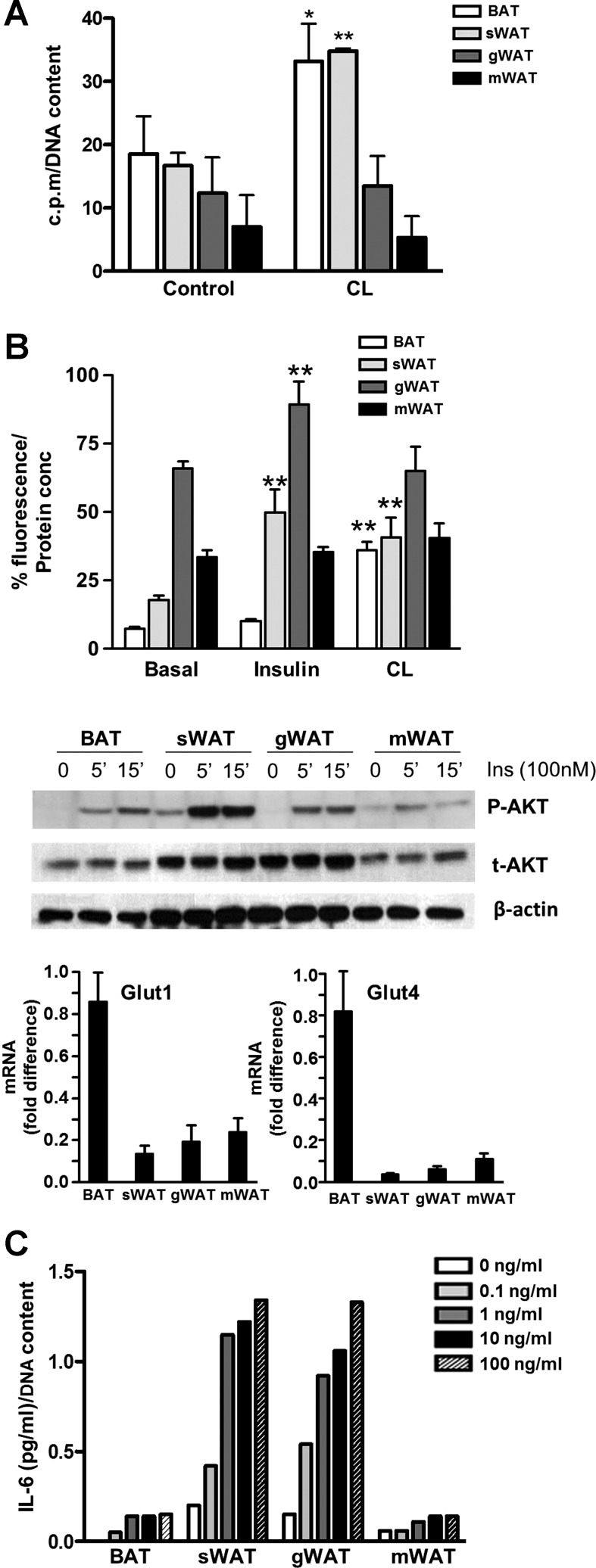
Functional characterization of immortalized cell lines from different adipose tissue depots. *A*: [^3^H]palmitate oxidation in differentiated adipocytes generated from interscapular BAT and sWAT, gWAT, and mWAT treated with or without CL-316,243 (CL; 10 μM) for 5 h. Results are expressed as a mean of 3 experiments and radioactivity count numbers normalized to DNA content. *B*: basal, insulin (100 nM), and CL (10 μM, 5 h) stimulated glucose uptake measured with the fluorescent glucose analog 2-[*N*-(7-nitrobenz-2-oxa-1,3-diazol-4-yl)amino]-2-deoxy-d-glucose. Results are expressed as a mean of 3 experiments and fluorescence normalized to protein content. p-Akt was measured by Western blot with antibody against phosphorylated Ser^473^. A representative blot is shown. GLUT1 and GLUT4 mRNA relative expression levels are shown in differentiated cells. *C*: IL-6 production by differentiated adipocytes after 24-h treatment with increasing amounts of LPS. Bars represent means ± SE, and significant differences are shown. *P < 0.05; **P < 0.01.

We also performed a series of functional metabolic assays to investigate whether the differences observed at the transcriptional level had physiological relevance in our cell lines. Basal β-oxidation was highest in BAT and subcutaneous WAT (Fig. 6*A*). Because cold activates BAT β-oxidation (65), we treated the cells with 10 μM CL-316,243 for 5 h. We detected an increase in the β-oxidation by BAT and subcutaneous WAT cells, whereas it remained unchanged in gonadal and mesenteric WAT cells. Basal glucose uptake, assessed with a fluorescent glucose analog (2-NBDG) in mature adipocytes, was the highest in gonadal WAT cells, followed by mesenteric and subcutaneous WAT and BAT (Fig. 6*B*). Considering the recent findings by PET scans, surprisingly, BAT had the lowest basal glucose uptake. This cannot be explained by the expression levels of the glucose transporters GLUT1 and GLUT4 that were present at higher levels in BAT compared with WAT cell lines (Fig. 6*B*) and is likely due to differences between white and brown adipocytes in the pathways controlling glucose uptake. Upon insulin stimulation, glucose uptake increased in gonadal and subcutaneous WAT cells by 30 and 20%, respectively, whereas CL-316,243 treatment increased BAT adipocyte glucose uptake (Fig. 6*B*), showing that β-adrenergic stimulus is more important than insulin for BAT glucose uptake. Furthermore, Akt phosphorylation was increased by insulin stimulation in all cell lines very potently in WAT and subcutaneous WAT, indicating that insulin signaling was intact in the differentiated adipocytes (Fig. 6*B*).

Adipocytes are an important source of inflammatory cytokines (34), and our microarray analysis revealed that WAT depots preferentially expressed gene pathways associated with inflammatory processes. Because previous investigations showed that differentiated adipocytes from human primary cultures were highly sensitive to LPS-stimulated secretion of IL-6 (11), we investigated the inflammatory response of differentiated adipocytes derived from distinct adipose tissues. Basal IL-6 secretion was detected in all cell lines except for BAT, and stimulation with LPS increased IL-6 production in a dose-dependent manner in subcutaneous and gonadal WAT cells (Fig. 6*C*). The lack of response of the mesenteric adipocytes was somewhat surprising given the link between visceral depots and inflammatory activity. Similar levels of TLR4 mRNA, the receptor for LPS, were detected in all cell lines following differentiation and induced >2,000-fold in all of the cell lines in differentiated compared with preadipocytes (data not shown).

This is the first time immortalized cell lines from four different adipose depots (interscapular BAT and subcutaneous, gonadal, and mesenteric WAT) have been generated and compared. Results demonstrate that it is possible to maintain depot-specific differences in expression and physiology in in vitro systems.

### Genes and Motifs Defining Browning of White Fat

**Fig. 7.**
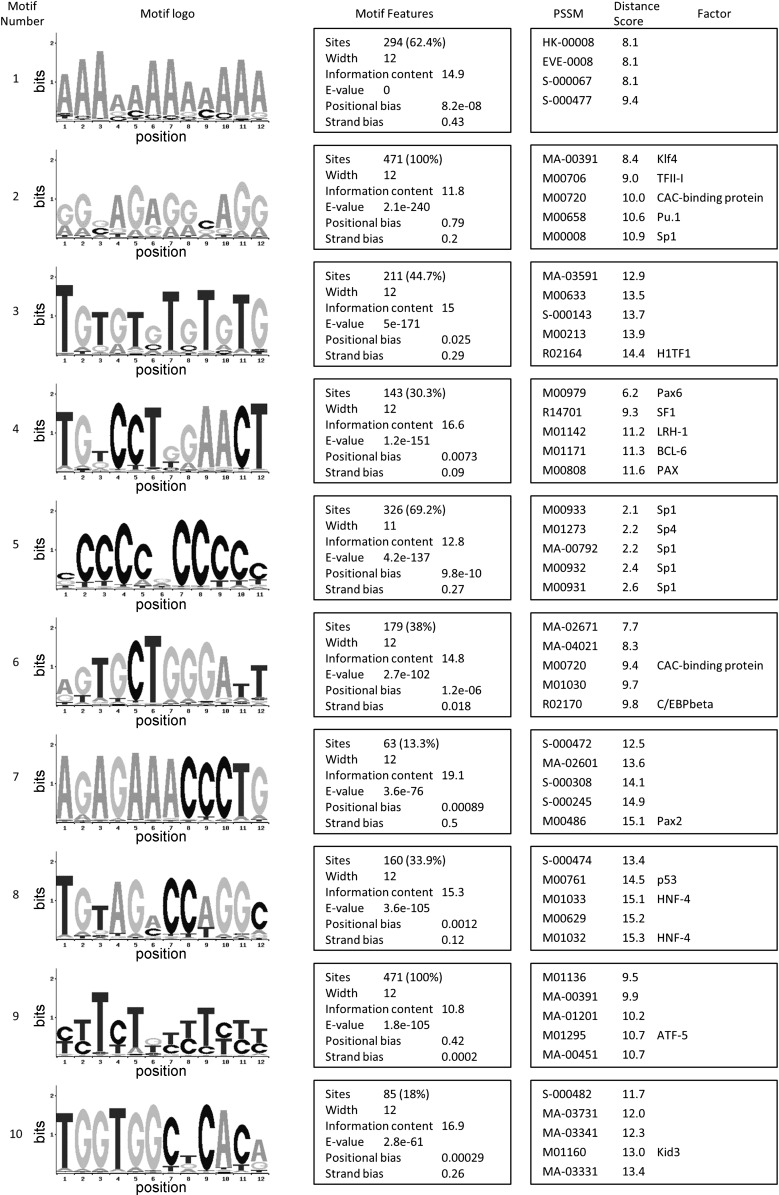
Motif analysis of brite gene promoters. Motif logos are presented from the MEME-LaB-analyzed gene cluster for transcripts induced by cold in subcutaneous WAT and more highly expressed in BAT vs. WAT depots. The MEME-LaB software determined 10 motifs, and these were analyzed for similarity to know motifs from JASPAR, PLACE, and TRANSFAC using position-specific scoring matrix (PSSM). Eukaryotic transcription factor binding sites are indicated for each relevant PSSM.

To characterize the transcription profile of WAT subject to browning in response to cold, we first prepared a list of genes that were enriched in both BAT vs. subcutaneous WAT and BAT vs. mesenteric WAT (comparisons at 28°C), which provides BAT genes. Next, we determined which genes from this list were also increased in subcutaneous WAT by cold exposure. The genes of this brite transcriptome are presented in Table 3. This stringent analysis confirmed the association of UCP1, PGC-1α, Cidea, Plin5, PPARα, and Otop1 with the browning of adipose tissue and also revealed additional genes that were part of the brown/brite transcription “fingerprint,” including GPR120, Nrg4, Rgs7, and Letm1. To investigate the regulatory motifs associated with brite genes, we took the 1,000-bp upstream flanking region from the top 308 sequences. After the removal of duplicates and rebuilding of mixed contigs, a total of 471 sequences were submitted to MEME-LaB. The 10 most significant motifs between six and 12 bp were identified and their similarity to known eukaryotic transcription factor-binding sites determined (Fig. 7). For motif 2, which was present in 100% of promoter sequences, the transcription factors Klf4, CAC-binding protein, Pu.1, and Sp1 were the nearest fit from their position-specific scoring matrix (PSSM). Other transcription factors that were identified as fitting the motifs included C/EBPβ, p53, Pax6, Pax2, BCL-6, ATF-5, Kid3 (Zfp354c), and the nuclear receptors SF1, LRH-1, and HNF-4. Examination of the microarray data reveals that C/EBPβ, BCL-6, SF1, Pax6, and ATF-5 are more highly expressed in brown vs. white adipose tissues at thermoneutrality. This highlights the potential of these factors through binding to their respective motifs to affect brown adipocyte gene expression.

**Table 3.** List of genes that define the “browning” of cold-activated white adipose tissue

**Mitochondria**	**Metabolism**	**Signaling**
Bckdha	Acaa2	2310014F07Rik
Cabc1	Acads	Adcy3
Chchdh10	Acadvl	Adora1
Coq3	Acc	Adrb3
Coq5	Acot11	Akap7
Coq9	Acot4	Angp13
Cox7a1	Acsf2	Angpt14
Cox8b	Cacb	Arl4a
Crat	Coasy	Gnao1
Cyc1	Cyp2b10	Gpr120
Decr1	Fbp2	Gpr135
Etfdh	Fn3k	Gpr146
Hsha	Gyk	Gpr4
Idh3a	Ldhb	Igsec2
Letm1	Pank1	Map2k5
Letmd1	Pck1	Mtor
Me3	Pdhb	Pde8a
Plat	Pdhx	Prkaca
Rilp	Pdk2	Prkar2b
Sirt3	Pdk4	Rgs7
Ucp1	Pfkb3	Adora1
Uqcr10	Sod2	**Immune/Inflammatory**
Uqcrfs1	**Transcription**	C8 g
**Transporters**	Esrra	Ifnar2
AI317395	Esrrg	Igsf21
Aqp7	Klf11	Il15ra
Cpt1	Nr1d1	Kng1
Cpt2	Nr1d2	Serpina12
Fabp3	Ppargc1a	**Lipid droplet**
Fabp4	Ppargc1b	Cidea
Slc27a2	Ppara	Cideb
Slc1a5	Sox6	Plin5
Slc25a20	Tef	Pnpla2
Slc25a33	Tfb2m	Lipe
Slc25a34	Tfdp2	**Cell cycle**
Slc25a42	Zfp703	Ccno
Slc4a4	**Cytoskeleton**	Cdkl3
Tmem37	Ka7na12	Inca1
**Hormone/vitamin**	Fam82b	**Apoptosis**
Cyp2b10	Tuba8	Nol3
Dio2	**Nucleic acid**	Otopn
Retsat	Asp	**Ion binding**
Rdn16	Carhsp1	Adprhl1
**Peroxisomes**	Gchfr	S100b
Hacl1	Gna13	Smyd4
Pex3	Oplah	Calr3
Pex19	Pn1dc1	**Unknown**
	Poln	4122401k19Rik
**Structural**	Satv2	8430408G22Rik
Krt79	**Secretion/vesicles**	9130214F15Rik
	Chpt1	9530008L14Rik
**Circadian rhythm**	Cpn2	Fam13a
Per3	Fam15a	Fam195a
**Protein degradation**	Otop1	Fam69b
Fbxo21	Pm20d1	
**Cell adhesion**	**Growth factor**	
Tspan18	Nrg4	

Genes listed were upregulated in subcutaneous WAT and more highly expressed in both BAT versus subcutaneous WAT and BAT versus mesenteric WAT.

Finally, the microarray data was analyzed to determine which transcripts were temperature regulated but not specifically associated with higher levels in BAT. We generated a list of genes that were more highly expressed at 6 vs. 28°C in subcutaneous WAT but not more highly expressed in BAT vs. mesenteric WAT or BAT vs. subcutaneous WAT (Table 4). Remarkably, of this list of 53 cold-induced genes in subcutaneous WAT, 40 were also cold-induced in BAT. Genes that were increased in both WAT and BAT in response to cold include Cil6l, Acaa1b, Dbp, Drd1a, Gys2, Lipf, Pla2g2e, and Sphk2. They are likely to be important for changes required only in response to cold and may be required for elevation of metabolic activity that will occur in both WAT and BAT in cold conditions. Although these genes are not more highly expressed in BAT vs. WAT at thermoneutrality, they may still be important for the brite program.

**Table 4.** Genes that are induced by cold in subcutaneous WAT and are not more highly expressed in BAT versus subcutaneous WAT or BAT versus mesenteric WAT

Gene Name	Gene	Induced by Cold in BAT	Gene Function
4921536K21Rik	RIKEN cDNA 4921536K21 gene	Yes	
**9030619P08Rik**	**RIKEN cDNA 9030619P08 gene**	Yes	
9430098F02Rik	RIKEN cDNA 9430098F02 gene	Yes	
Abca6	ATP-binding cassette, sub-family A (ABC1), member 6		
**Abhd1**	**Abhydrolase domain containing 1**	Yes	
**Acaa1b**	**Acetyl-Coenzyme A acyltransferase 1B**	Yes	
Acoxl	Acyl-coenzyme A oxidase-like		
Adrm1	Adhesion regulating molecule 1	Yes	
**Apoc2**	**Apolipoprotein C-II**	Yes	Extracellular
Apoc4	Apolipoprotein C-IV		Extracellular
**Apold1**	**Apolipoprotein L domain-containing 1**	Yes	
Aqp11	Aquaporin 11	Yes	Plasma membrane
Atrn	Attractin	Yes	Plasma membrane,
C2cd2l	C2 calcium-dependent domain containing 2-like	Yes	
Catsper3	Cation channel, sperm associated 3		Plasma membrane
Ccny	Cyclin Y	Yes	
**Chrna2**	**Cholinergic receptor, nicotinic, alpha polypeptide 2**	Yes	Plasma membrane
Cir1	Corepressor interacting with RBPJ, 1		
**Cldn22**	**Claudin 22**		Plasma membrane
Cmbl	Carboxymethylenebutenolidase-like (Pseudomonas)		
**Crtac1**	**Cartilage acidic protein 1**		Extracellular
**Cyp4a10**	**Cytochrome P450, family 4, subfamily a, polypeptide 10**	Yes	
**Dbp**	**D site albumin promoter binding protein**	Yes	Transcription factor
Dcst1	DC-STAMP domain containing 1		
**Drd1a**	**Dopamine receptor D1A**	Yes	Signaling Molecule
Fabp12	Fatty acid-binding protein 12	Yes	
Fndc8	Fibronectin type III domain containing 8	Yes	
**Gys2**	**Glycogen synthase 2**	Yes	
Katna1	Katanin p60 (ATPase-containing) subunit A1	Yes	Cell Cycle
Klhl2	Kelch-like 2, Mayven (Drosophila)		
Kras	v-Ki-ras2 Kirsten rat sarcoma viral oncogene homolog	Yes	Signaling Apoptosis
**Lipf**	**Lipase, gastric**		Extracellular
Lsm1	LSM1 homolog, U6 small nuclear RNA associated	Yes	
Mfsd3	Major facilitator superfamily domain containing 3	Yes	
Mpv17l	Mpv17 transgene, kidney disease mutant-like	Yes	
Nploc4	Nuclear protein localization 4 homolog (S. cerevisiae)	Yes	
Nxnl1	Nucleoredoxin-like 1	Yes	
Padi3	Peptidyl arginine deiminase, type III		
Pex16	Peroxisomal biogenesis factor 16	Yes	
**Pla2 g2e**	**Phospholipase A2, group IIE**	Yes	Extracellular
Psmd4	Proteasome (prosome, macropain) 26S subunit, nonATPase	Yes	
Rcl1	RNA terminal phosphate cyclase-like 1		
Reep6	Receptor accessory protein 6	Yes	
Rnf146	ring finger protein 146	Yes	
Rora	RAR-related orphan receptor alpha	Yes	Transcription factor
Slc9a6	Solute carrier family 9 member 6	Yes	
**Sphk2**	**Sphingosine kinase 2**	Yes	Signaling molecule
**Tbata**	**Thymus, brain and testes associated**		
**Tinag**	**Tubulointerstitial nephritis antigen**	Yes	
Tmem120a	Transmembrane protein 120A	Yes	
Trfr2	Transferrin receptor 2	Yes	Plasma membrane
**Vmn2r1**	**Vomeronasal 2, receptor 2**	Yes	
Zwint	ZW10 interactor	Yes	Cell cycle

Boldface indicates genes that are within the top 200 most significantly upregulated genes in 6°C versus 28°C subcutaneous WAT.

### Nrg4 is a Brown Adipocyte Adipokine That Promotes Neurite Outgrowth

**Fig. 8.**
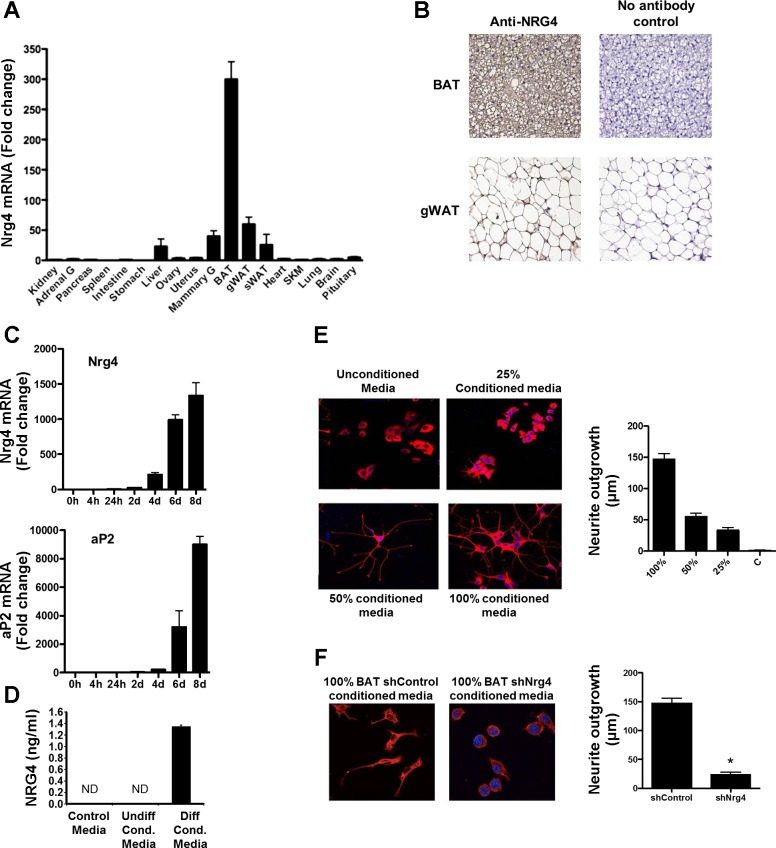
Nrg4 is a BAT adipokine promoting neurite outgrowth. *A*: Nrg4 mRNA expression in mouse tissues (*n* = 4). Data are expressed as a fold difference compared with kidney. *B*: inmunohistochemistry of Nrg4 in murine BAT and gonadal WAT. *C*: Nrg4 and aP2 mRNA expression in immortalized BAT cells throughout differentiation. Data are shown as fold induction compared with *time 0*. *D*: NRG4 levels in unconditioned medium (control), conditioned medium from undifferentiated brown adipocytes (Undiff Cond), and 4-wk-differentiated brown adipocytes (Diff Cond) in 6-well plates, determined by ELISA. DNA content was 28.65 ± 1.38 and 26.04 ± 1.96 μg/well for differentiated and undifferentiated cells, respectively. *E*: neurite staining with anti-Tuj1 antibody of PC12-HER4 cells incubated with different concentrations of conditioned medium from differentiated brown adipocytes. Neurite outgrowth measured in μm and expressed as fold induction over control cells. *F*: neurite staining by anti-Tuj1 antibody of PC12-HER4 treated with conditioned medium of differentiated brown adipocytes stably expressing shNrg4 or a nontargeting shRNA. For all of the data, bars represent means ± SE, and significant differences are shown. *P < 0.05.

Nrg4 was one of the genes for the group that define the brite transcription signature and was selected for further study. It showed an expression pattern similar to the BAT marker gene Cidea in that expression was greater in BAT vs. WAT and that cold stimulated a large increase in WAT, whereas only a small increase was detected in BAT. Along with Nrg1, -2, and -3, it is a member of the epidermal growth factor family of proteins that are involved in normal development and in the pathology of many diseases. Analysis of a panel of mouse tissues revealed that Nrg4 was highly expressed in adipose tissues, being the highest in BAT, followed by gonadal and subcutaneous WAT (Fig. 8*A*). Analysis of in vivo protein expression by immunohistochemistry revealed the presence of NRG4 in BAT and gonadal WAT (Fig. 8*B*). For in vitro studies, we determined expression of Nrg4 mRNA in our immortalized BAT cell line. Expression progressively increased upon differentiation treatment, being the highest after 8 days (Fig. 8*C*), confirming adipocyte-specific expression (Fig. 5*C*). Efficient differentiation was confirmed by the induction of the adipose marker aP2 (Fig. 8*C*). To test whether NRG4 was secreted into the culture media and whether this had any effect in promoting neurite outgrowth, conditioned medium of mature brown adipocytes was used to treat PC12 cells stably expressing ErbB4 (25). NRG4 was detected by ELISA in conditioned medium from differentiated brown adipocytes and was undetectable in unconditioned medium and medium from preadipocytes (Fig. 8*D*). Conditioned medium induced neurite outgrowth (measured by staining with anti-Tuj1 antibody) after 24 h in a dose-dependent manner (Fig. 8*E*). This effect was specific for Nrg4, as neurite outgrowth was prevented in conditioned medium from adipocytes in which it was knocked down by >75% by shRNA interference (Fig. 8*F*). Thus, we can conclude that NRG4 produced by brown adipocytes in culture can affect the neurite development in neural cells. This indicates that NRG4 represents a novel brown adipose tissue adipokine with important autocrine/paracrine functions in the development of the innervation net in adipose tissues, with implications in the acquisition of BAT features in WAT depots.

## DISCUSSION

In the present study, we aimed to identify factors, pathways, and regulatory elements that are important for acquiring brown fat features in WAT depots. We conducted global expression analysis on discrete adipose tissue depots, with well-recognized differential physiology and involvement in metabolic disease, in mice that had been exposed to cold for 10 days. This stimulus drives the appearance of brown adipocytes or the browning of determined WAT depots. Our results support previous findings that the subcutaneous adipose depot is most reactive to acquiring BAT characteristics, whereas visceral depots are much less responsive (28). Female gonadal WAT shows a certain degree of induced expression of UCP1, PGC-1α, Cidea, and other BAT-specific genes upon adrenergic activation triggered by cold exposure. Mesenteric WAT seems to be almost completely insensitive at the level of gene expression, although interestingly, adipocytes here become multilocular, albeit UCP1-ve, in response to cold (40). In contrast, BAT shows the highest number of genes that are affected upon cold exposure. We have also identified a set of genes and pathways that are either induced or repressed by the tissue remodeling that occurs after the long-term adrenergic activation induced by cold exposure. VEGF signaling was identified as downregulated by cold exposure, with Vegfc specifically reduced and also more highly expressed in WAT compared with BAT. This contrasts with Vegfa, which was found to be present at higher levels in BAT. This agrees with previous reports that associate Vegfa (64) with angiogenesis in BAT and Vegfc being negatively regulated by adrenergic signaling (5) and indicates distinct roles for Vegfc and Vegfa signaling in white and brown adipocytes, respectively.

We have highlighted a set of genes, the expression of which can be induced by these signaling events. These include Bmp8b, a member of the TGFβ family that has recently been found to be involved in brown adipose tissue activation (62); Nrg4, a member of the neuregulin family involved in neurite growth (23); GPR120, a GPCR that has been shown to mediate the anti-inflammatory and insulin-sensitizing effects of ω-3 fatty acids, with its absence making mice prone to obesity (29); Rgs7, a regulator of G protein signaling that can inhibit signal transduction by increasing the GTPase activity of G protein-α subunits (4); Slc27a2, an isozyme of the long-chain fatty acid-coenzyme A ligase family that has the capacity to convert free long-chain fatty acids into fatty acyl-CoA esters and also function as a fatty acid transporter (26); Letm1 a mitochondrial protein involved in calcium transport (60); and Nnat, a member of the proteolipid family of amphipathic polypeptides that exhibits paternally inherited imprinted expression and is considered to play an important role in brain development (33) and glucose-mediated insulin secretion (32). For some, this is the first time that expression has been described in adipocytes. More importantly, with our strategy, we have been able to identify genes that have implications in metabolic pathophysiology in mouse and/or humans, including GPR120 (29), Rgs7 (2), and Nnat (58), that have been reported to be associated with human obesity in linkage and single-nucleotide polymorphism analysis. Further studies will address the relevance of these molecules for adipocyte/adipose tissue functionality.

We have compared the transcriptional differences in our microarray with other available data sets. In the present study, because intact females were used, some genes may be subject to cyclical hormonal changes. Although there were a number of technical differences between studies, including using the C57BL/6 strain, the use of males, and maintenance at 23°C [GEO accession no. GSE44059 (44)], the fact that the same microarray platform was used and expression values were available for exactly the same probes allowed us to perform a direct comparison of the differentially expressed transcripts. This was conducted in OrderedList, a package in R, for meta-analysis of ordered gene lists like those acquired from differential gene expression analysis (37). The *P* value for a significant overlap between the two expression studies being substantially different from random studies is <10^−16^. Nrg4, Slc27a2, Rgs7, and Nnat were differentially expressed in the GSE44059 data sets for BAT vs. inguinal WAT and BAT vs. epididymal WAT. The same genes were also differentially regulated in an independent microarray of BAT vs. epididymal WAT [GEO accession no. GSE8044 (48)]. Our experiments were performed at thermoneutrality, which may reduce the baseline expression of BAT genes in WAT, thereby allowing the identification of additional cold-regulated genes such as GPR120 and Bmp8b, which were not differentially expressed in the other data sets.

In contrast to BAT-enriched genes, the data sets are remarkably divergent for genes that are more highly expressed in WAT compared with BAT. Upk3b showed the greatest difference in expression between BAT and Mes WAT in our study at 28°C and was found in GSE44059 (BAT vs. epididymal WAT) and GSE8044 (BAT vs. subcutaneous WAT). Similarly, Nnat was also found in these analyses. Other genes consistently elevated in WAT compared with BAT across the different data sets include Sncg, Ighv14-2, Ildr2, Samsn1, and Synpo2.

With regard to other analyses of cold-dependent changes in gene expression in inguinal WAT, the genes we have identified are highly consistent with a comparison of a 5-wk exposure to 4 vs. 30°C male C57BL/6 mice [GEO accession GSE13432 no. (64)]. Importantly, Nrg4, GPR120, Slc27a2, Rgs7, Letm1, Bmp8b, and Cil6l (9030619P08Rik) were also cold induced in this microarray. In contrast, there was little consistency in the genes more highly expressed at 30 vs. 4°C in the independent experiments. It is noteworthy that Nnat was one of the few genes that was found in both microarrays (Acin1, Ccr2, Far1, Pida3, Zfp36l, Tnfrsf21, and Ostc were also more highly expressed at thermoneutrality in the 2 analyses).

The consistency across our microarray, GSE44059, GSE8044, GSE13432, and other published studies confirms the expression profile of already identified BAT and brite genes implicated in transcriptional regulation (Zic1, Lhx8, PGC-1α, and Prdm16), mitochondrial function (Cox7a1, UCP1), lipid metabolism (Gyk, Fabp3, Plin5, Elovl3, Pank1, and Cidea), cytokine signaling (Otop1), enzymatic activity (Dio2, Cpn2), and coagulation regulation (Kng1).

It is transcriptional regulators that will be pivotal to expression of the brown fat phenotype. Our analysis of relevant controls showed PGC-1α and PRDM16 were more highly expressed in BAT compared with WAT depots, and the subcutaneous WAT depot showed increased levels of both factors in response to cold exposure. Therefore, these transcriptional regulators or their interplay with coregulators such as RIP140, which at 4°C was lower in BAT vs. WAT, may be central to the changes in gene expression due to cold or the depot-specific profiles. Further work is necessary to determine the role of other factors such as Zic1 and Lhx8 in brown and brite gene expression.

We sought to identify regulatory motifs that were specifically enriched in the promoters of the cluster of brite genes found from the microarray. Analysis using the MEME-LaB software determined the association of Sp1-binding sites with one of the motifs. This transcription factor may be relevant to induction of BAT genes due its interaction with PPARγ (1, 52). Other notable transcription factors that had putative binding sites enriched in the promoters of the brite cluster include p53 and C/EBPβ, which are known regulators of BAT differentiation (35, 38). HNF-4 has recently been reported to be downregulated in WAT by cold, and therefore, it may serve to repress BAT gene expression in this tissue (50). Other factors with putative binding sites, such as BCL-6, SF1, Pax6, and ATF-5, showed elevated expression in BAT compared with WAT, and therefore, they represent novel potential regulators of the brown or brite gene expression pattern.

It is well established that adipose tissues are composed of lipid-filled mature adipocytes and other nonadipocyte cells, which form the SVF. The SVF consists of various types of cells, including immune cells, fibroblasts, pericytes, endothelial cells, adipocyte progenitors, and stromal cells as well as undefined pools of stem cells. Fractionation of the different depots showed that all of the genes that were selected from the microarray for validation or further study were enriched in adipocytes compared with the SVF. To evaluate isolated adipocytes from discrete depots, we generated cell lines from precursors of four different adipose depots (BAT and subcutaneous, gonadal, and mesenteric WAT). Our immortalization protocol, which involves the inactivation by temperature of the SV40 T antigen prior to differentiation, prevents sequestration of retinoblastoma protein, avoiding the risk of driving white preadipocytes into “brown-like” adipocytes (45). The adipocyte cell lines demonstrate clear characteristics that correspond to their depot of origin, with the highest expression of UCP1, Cidea, Rgs7, and Nrg4 detected in the differentiated brown adipocytes. Cil6l was present at the highest levels in differentiated subcutaneous white adipocytes, in agreement with tissue expression pattern. The induction of UCP1 and PGC-1α, in response to a β_3_-stimulus, was greatest in the adipocytes generated from BAT, followed by subcutaneous WAT and gonadal WAT, with mesenteric WAT being unresponsive. The cell lines we generated retain functional β-adrenergic and insulin pathways, oxidative metabolism, and an inflammatory response that very similar to what is found in the different adipose tissue depots. Overall, we can conclude that these cell lines are a reliable model for in vitro studies of different adipose tissue depots and are a powerful tool for future drug discovery studies.

We undertook further study of Nrg4, as it was both highly enriched in BAT vs. WAT and upregulated in WAT following cold exposure and seemed likely to be an important novel signaling factor in adipose tissue that could have a key role in adipocyte-neuronal cross-talk. We found NRG4 to be secreted by brown adipocytes but not preadipocytes. Using PC12 pheochromocytoma cells stably expressing the receptor for NRG4 and ERBB4, we have been able to show that NRG4 is a BAT/brite adipokine. One of the key differences between brown and white adipose tissue is the degree of sympathetic innervation (54). Previous studies have indicated that nerve growth factor is produced in BAT (41). Our data indicate that NRG4 could be a key factor for the development of BAT and acquisition of BAT features of WAT depots by promoting the growth of neurites and thus enhancing innervation of the tissue needed to activate the thermogenic functions. To fully evaluate the role of Nrg4 in the regulation of adipose tissue innervation, further studies will require the generation of mouse models with depot-specific ablation of the gene.

The discovery that adult humans possess functional BAT has opened the possibility for future treatments against obesity that would boost BAT activity or promote the appearance of brown adipocytes in nonclassical BAT locations where white fat predominates by BAT-inducing drugs or other therapies, such as tissue transplantation or gene therapy. The identification of the key transcriptional events associated with browning of WAT is important for the understanding of how gene expression governs this tissue remodeling.

## GRANTS

This work was supported by Biotechnology and Biological Sciences Research Council Grant BB/H020233/1, the EU FP7 project DIABAT (HEALTH-F2-2011-278373), Wellcome Trust Grant WT093082MA, and the Genesis Research Trust.

## DISCLOSURES

The authors declare that they have no conflicts of interest, financial or otherwise.

## AUTHOR CONTRIBUTIONS

M.R., A.F., J.O.T., S.C., M.G.P., and M.C. conception and design of research; M.R., A.F., A.O., E.N., S.M., M.E.F., D.M., E.B., W.J.G., S.C., and M.C. performed experiments; M.R., M.K., A.F., A.O., Y.-W.C., E.N., S.M., M.E.F., D.M., J.D.M., W.J.G., G.M., and M.C. analyzed data; M.R., J.D.M., G.M., M.G.P., and M.C. interpreted results of experiments; M.R., M.K., A.F., A.O., Y.-W.C., and M.C. prepared figures; M.R. and M.C. drafted manuscript; M.R., S.C., M.G.P., and M.C. edited and revised manuscript; M.R., J.D.M., and M.C. approved final version of manuscript.
